# Comparison of Classification Methods for P300 Brain-Computer Interface on Disabled Subjects

**DOI:** 10.1155/2011/519868

**Published:** 2011-09-18

**Authors:** Nikolay V. Manyakov, Nikolay Chumerin, Adrien Combaz, Marc M. Van Hulle

**Affiliations:** Laboratorium voor Neuro- en Psychofysiologie, K.U.Leuven, Campus Gasthuisberg, O&N 2, Bus 1021, Herestraat 49, B-3000 Leuven, Belgium

## Abstract

We report on tests with a mind typing paradigm based on a P300 brain-computer interface (BCI) on a group of amyotrophic lateral sclerosis (ALS), middle cerebral artery (MCA) stroke, and subarachnoid hemorrhage (SAH) patients, suffering from motor and speech disabilities. We investigate the achieved typing accuracy given the individual patient's disorder, and how it correlates with the type of classifier used. We considered 7 types of classifiers, linear as well as nonlinear ones, and found that, overall, one type of linear classifier yielded a higher classification accuracy. In addition to the selection of the classifier, we also suggest and discuss a number of recommendations to be considered when building a P300-based typing system for disabled subjects.

## 1. Introduction

Research on *brain-computer interfaces* (BCIs) has witnessed a tremendous development in recent years [1] that has even been covered in the popular media. Although a lot of research has been done on invasive BCIs, leading to brain implants decoding neural activity directly, which are primarily tested on animals, noninvasive BCIs, for example, based on *electroencephalograms* (EEG) recorded on the subject's scalp, have recently enjoyed an increasing visibility since they do not require any surgical procedure, and can therefore be more easily tested on human subjects. Several noninvasive BCI paradigms have been described in the literature, but the one we concentrate on relies on *event-related potentials* (ERPs, a stereotyped electrophysiological response to an internal or external stimulus [2]). 

One of the most explored ERP components is the P300. It can be detected while a subject is shown two types of events with one occurring much less frequently than the other (“rare event”). The rare event elicits an ERP consisting of an enhanced positive-going signal component with a latency of about 300 ms after stimulus onset [2]. In order to detect ERPs, single-trial recordings are usually not sufficient, and recordings over several trials need to be averaged: the recorded signal is a superposition of the activity related to the stimulus and all other ongoing brain activity together with noise. By averaging, the activity that is time locked to a known event (e.g., the onset of the attended stimulus) is extracted as an ERP, whereas the activity that is not related to the stimulus onset is expected to be averaged out. The stronger the ERP signal, the fewer trials are needed, and *vice versa*.

There has been a growing interest in the ERP detection problem, as witnessed by the increased availability of BCIs that rely on it. A notorious example is the P300 speller [3], with which subjects are able to type words on a computer screen. This application meets the BCI's primary goal, namely, to improve the quality of life of neurologically impaired patients suffering from pathologies such as amyotrophic lateral sclerosis (ALS), brain stroke, brain/spinal cord injury, cerebral palsy, muscular dystrophy, and so forth. But, as it is mostly the case with BCI research, the P300 BCI has primarily been tested on *healthy* subjects. Only very few attempts have been made on *patients* [4–9]. Several of these tests on patients [4, 9] deal with P300-based online typing, however, since only very few patients were tested, it is still an open question for which patient categories the P300 speller is best suited. 

In addition, the performances of different P300 classifiers were compared for healthy subjects only, and their outcomes were found to disagree to some extent. In [10], a comparison of several classifiers (Pearson's correlation method, Fisher's linear discriminant analysis (LDA), stepwise linear discriminant analysis (SWLDA), linear support-vector machine (SVM), and Gaussian kernel support vector machine (nSVM)) was performed on 8 healthy subjects. It was shown that SWLDA and LDA render the best overall performance. In [11], it was shown that, among linear SVM, Gaussian kernel SVM, multi-layer perceptron, Fisher LDA, and kernel Fisher Discriminant, the best performance was achieved with LDA. Based on these studies, albeit different sets of classifiers were used, one can conclude that linear classifiers work better than nonlinear ones, at least for the case of the P300 BCI on healthy subjects. This statement is also supported by other researchers (e.g., in [12]).

In light of this, and since a classifier comparison has never been performed on patients, it remains an open question what is the best classifier in this case. This is indeed an important question since the P300 responses from healthy subjects and patients can be quite different [5]. Thus, the outcome of a comparison for healthy subjects might not be valid for patients. 

In this paper, we report on tests performed on a group of (partially) disabled patients suffering from amyotrophic lateral sclerosis (ALS), middle cerebral artery (MCA) stroke, and subarachnoid hemorrhage (SAH). In addition to the classifiers mentioned above, we also add two more linear ones (i.e., Bayesian linear discriminant analysis and a method based on feature extraction), since they have been used before in P300 BCIs [7, 13]. In summary, we compare a more extensive set of classifiers and perform our comparison on patients, instead of on healthy subjects, both of which distinguish our study from others.

## 2. Methods

### 2.1. EEG Data Acquisition

**Figure 1:**
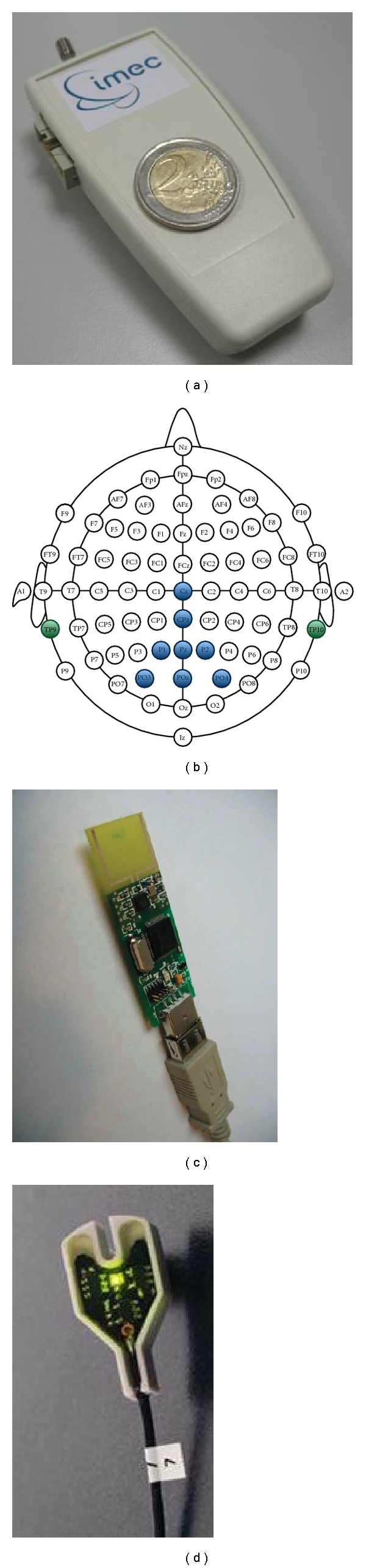
(a) Wireless 8 channel amplifier. (b) Locations of the electrodes on the scalp. (c) USB stick receiver. (d) Active electrode.

Our recordings were performed with a prototype of a* miniature* EEG recording device that * wirelessly* communicates with a USB stick receiver (Figures 1(a) and 1(c)). The prototype was developed by *imec *(http://www.imec.be/) and built around their ultra-low power 8-channel EEG amplifier chip [14]. The EEG data were recorded at a sampling frequency of 1000 Hz, which is fixed by the hardware. A laptop working under Windows XP SP3 with a bright 15′′ screen was used for the visual stimulation as well as for EEG data recording, processing and storing. 

We used an electrode cap with large filling holes and sockets for active Ag/AgCl electrodes (ActiCap, Brain Products, Figure 1(d)). The eight electrodes were placed primarily on the parietal pole, namely at positions Cz, CPz, P1, Pz, P2, PO3, POz, and PO4, according to the international 10–10 system (Figure 1(b)). The reference and ground electrodes were placed on the left and right mastoids, respectively.

Each experiment started with a pause of approximately 90 s, which is required for the EEG amplifier to stabilize its internal filters. During this period, the EEG signals were not recorded. The data for typing each character (see Section 2.3 for details) were recorded in one session. As the duration of each session is known *a priori*, as well as the data transfer rate, it is easy to estimate the amount of data transmitted during a session. We used this estimate, increased by a 10% margin, as the size of the serial port buffer. To make sure that the entire recording session for one character fits completely into the buffer, we cleared the buffer just before recording. This strategy allowed us to avoid broken/lost data frames, which might occur due to a buffer overflow. The EEG data frames were only in rare cases lost during wireless transmission: under normal experimental conditions, the data loss is negligible (<0.01%) and never more than a few consecutive samples, which could be (linearly) reconstructed from the successfully received ones. The amount of broken/lost frames can be precisely computed using the counter incorporated into each data frame. 

### 2.2. Data-Stimuli Synchronization

Unlike a conventional EEG system, the system we used does not have any external synchronization inputs. We used a synchronization scheme based on the high-precision timestamps of the stimulus onsets (during stimulation). The timestamps are obtained using the high-resolution system performance counter (via QueryPerformanceCounter system call), which allows to achieve microsecond resolution. We save the timestamps of the EEG acquisition session start and end, as well as the timestamps of the stimulus onsets and offsets. Due to the fact that the EEG signal has a constant sampling rate, and assuming a constant (virtual) serial port latency, the precise mapping between the timestamps and the corresponding EEG data samples is straightforward. We used this mapping for partitioning the EEG signal into signal segments, for further processing. To eliminate the unwanted load of the computer system, all recordings were done on a PC working in a special (“experimental”) hardware profile, which has a minimal set of running services and devices. Additionally, we have raised the priority of the application, responsible for the visual stimulation and EEG data acquisition/processing, to the “high” level.

### 2.3. Experiment Design

Twelve subjects, naïve to BCI applications, participated in the experiments (ten male and two female, aged 37–66 with an average age of 51.25). The subjects were suffering from different types of brain disorders. The experimental protocol was approved by the ethical committee. After the recordings were made, four subjects were excluded from the classifier comparison, since their performance was close to chance level, possibly due to the nature of their brain disorder, or because they did not properly understand the experiment or were too tired to perform the task. The information about the patients, of which the recordings were further considered, that is, their diagnosis, age, and gender, is presented in Table 1.

**Figure 2:**
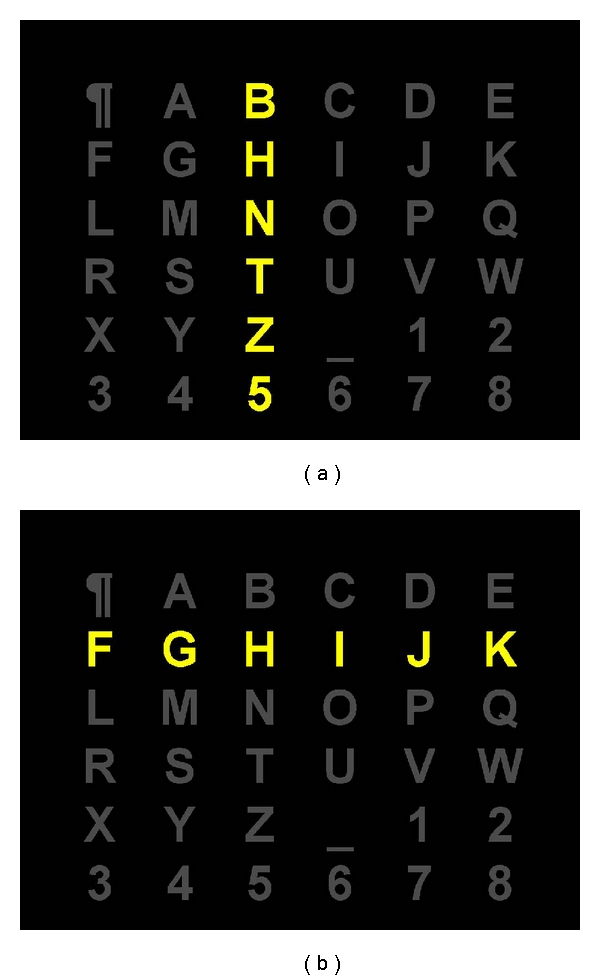
Typing matrix of the P300 speller. Rows and columns are intensified in random order; one trial consists of the intensifications all six rows and all six columns. The intensification of the third column (a) and of the second row (b) are shown.

We have used the same visual stimulus paradigm as in the first P300-based speller, introduced by Farwell and Donchin in [3]: a matrix of 6 × 6 characters. The only difference is with the character set: in our case, it is the usual set of 26 Latin characters, eight digits, but with two special characters (“_” instead of *space* and “*¶*” as the *end of input* indicator). Additionally, for some subjects, the Cyrillic alphabet was used. Each experiment was composed of one training and several testing stages. During both stages, columns and rows of the matrix were intensified (see Figure 2) in a random manner. The duration of the intensification was fixed to 100 ms, followed by 100 ms of no intensification. Each column and each row flashed only once during one trial, so each trial consisted of 12 stimulus presentations.

During the training stage, 11 characters, taken from the typing matrix, were presented to the subject. For each character, 10 intensifications for each row/column were performed. The subjects were asked to mentally count the number of intensifications of the intended character. The counting was used only to ensure that the participants paid attention. 

The recorded data was filtered (in the 0.5–15 Hz frequency band with a fourth-order zero-phase digital Butterworth filter) and cut into signal segments. Each of these segments consisted of 1000 ms of recording, starting from the stimuli onsets. Then, they were downsampled, by retaining every 25th sample, and assigned to one of two possible groups: *target* and *nontarget*, according to the stimuli that they were locked to. For training the classifier, we constructed a set of 1000 target, and the same amount of nontarget averaged brain responses, where the averages were taken based on *k* randomly selected responses from the corresponding groups in the training set. The number *k* was equal to the number of intensification sequences (trials), for each stimulus, during the testing stage.

Signal amplitudes at specific time instants in the interval 100–750 ms after stimuli onset, of the downsampled EEG signal, were taken as features. All these features were normalized to their *Z* score through the estimation of $f_{n,t}=(x_{n}(t)-\bar{x_{n}(t)})/\sigma_{x_{n}(t)}$, where *x*_*n*_(*t*) is the EEG amplitude of *n*th channel (electrode) at time *t*, after stimulus onset, $\bar{x_{n}(t)}$ the average of *x*_*n*_(*t*), and *σ*_*x*_*n*_(*t*)_ the standard deviation for all training examples of both the target and nontarget recordings of the training set. When combining all those features, we obtained a feature vector **f** = [*f*_1_,…, *f*_*N*_]^*T*^, which was used as input to either the linear classifier *w*_1_ *f*_1_ + *w*_2_ *f*_2_ + ⋯+*w*_*N*_ *f*_*N*_ + *b* = **w** ^*T*^ **f** + *b* (see further) or the nonlinear one, *y*(**f**, **w**, **b**). Since we use *Z* scores as features, and since we use a balanced training set (equal numbers of target and nontarget responses), the parameter *b* should be close to zero. After substituting the feature vector **f** into the above-mentioned equation, we obtain a “distance” from the point in feature space to the boundary (hyperplane in the linear case), separating the target from the nontarget class, with the sign indicating to which class the point belongs. 

After training the classifier, each subject performed several *online* test sessions during which (s)he was asked to *mind-type* a few words. In the case of a mistyping, (s)he was instructed to type further without trying to correct the mistake (“backspace” was not allowed). The typing performance (ratio of correctly typed characters) was used for estimating the classification accuracy. For these online test sessions, we considered a linear SVM classifier trained on data averaged over 15 trials. Thus, each subject attempted to type characters based on 15 row/column intensifications. About 36 characters were typed by each subject. This number slightly varied between subjects, since some subjects chose the characters to spell themselves (free spelling). During typing, the EEG data was stored for further (*offline*) analysis based on a smaller amount *k* of trials (in this case we used all *k*-combination of 15 trials for each typed character, for assessing the accuracy).

The testing stage differs from the training stage by the way the signal segments were grouped. During training, the system “knows” exactly which one of 36 possible characters is attended by the subject at any moment of time (copy spelling). Based on this information, the collected signal segments can be grouped into only two categories: target (attended) and nontarget (not attended). However, during testing, the system does not know which character is attended by the subject, and the only meaningful way of grouping is by stimulus type (which in the proposed paradigm can be one of 12 types: 6 rows and 6 columns). Thus, during the testing stage, for each trial, we had 12 segments (from all 12 types) of 1000 ms EEG data recorded from each electrode. The averaged EEG response for each electrode was determined for each stimulus type. The selected features of the averaged data were then fed into the classifier (see Section 3). As a result, the classifier produces 12 (for each row/column) values (*c*_1_,…, *c*_12_) which describe the distance to the class boundary in the feature space, together with the sign. The row index *i*_*r*_ and the column index *i*_*c*_ of the classified character were calculated as

(1) $$i_{r}=\arg\;\underset{i=1,\ldots ,6}{\max\;}\;\left\{c_{i}\right\},\;\;i_{c}=\arg\;\underset{i=7,\ldots ,12}{\max\;}\;\left\{c_{i}\right\}-6.$$

The character at the intersection of the *i*_*r*_^*th*^ row and *i*_*c*_^*th*^ column in the matrix was then taken as the result of the classification and presented, as a feedback, to the subject, in online mode. 

## 3. Classification Methods

### 3.1. Fisher's Linear Discriminant Analysis

Fisher's linear discriminant analysis (LDA) is one of the most widely used classifiers in P300 BCI systems [10, 15]. It was reported to even outperform other classifiers [11]. Its main idea is to find a projection from the *N*-dimensional feature space onto a one-dimensional space **w** ^*T*^ **f** for which the ratio of the variance between the two classes (target and nontarget) versus the variance within the classes is maximal. This “optimal” projection is estimated as **w** = (Σ_−1_ + Σ_+1_)^−1^(*μ*_+1_ − *μ*_−1_), where Σ and *μ* define the covariances and the means of the two classes (target and nontarget) that need to be separated.

### 3.2. Stepwise Linear Discriminant Analysis

Stepwise linear discriminant analysis (SWLDA) has been used in patient studies of the P300 BCI speller [4, 5]. It can be considered as an extension of the LDA with an incorporated filter feature selection. SWLDA adds and removes terms from a linear discriminant model, based on their statistical significance in regression, thus, producing model that is adjustable to the training data. It was shown that SWLDA performs equally well or even better than several other classification methods in P300 BCIs [10]. For our comparison, we have used the same procedure as in [10] (in the forward step, the entrance tolerance *P*-value < 0.1; in the backward step, the exit tolerance *P*-value > 0.15). The process was iterated until convergence, or until it reached a predefined number of 60 features.

### 3.3. Bayesian Linear Discriminant Analysis

Bayesian linear discriminant analysis (BLDA) has been used in P300 BCI patient studies [7]. It is based on a probabilistic regression network. Suppose that the targets *t*_*i*_ (in the case of a classification problem these are +1 and −1) are linearly dependent on the observed features **f** ^*i*^ = [*f*_1_^*i*^,…, *f*_*N*_^*i*^]^*T*^ with an additive Gaussian noise term *ε*_*n*_: *t*_*i*_ = **w** ^*T*^ **f** ^*i*^ + *ε*_*i*_. Assuming further an independent generation of the examples from a data set, the likelihood of all data is *p*(**t** | **w**, *σ* ^2^) = ∏_*i*=1_^*N*^(2*πσ* ^2^)^−1/2^exp (−(*t*_*i*_ − **w** ^*T*^ **f** ^*i*^)^2^/2*σ* ^2^). Additionally to this, we have to introduce a prior distribution over all weights as a zero-mean Gaussian

(2) $$p\left(w\;|\;\alpha \right)=\overset{n}{\underset{j=1}{\prod }}{\left(\frac{\alpha }{2\pi }\right)}^{1/2}\exp\;\left(-\frac{\alpha }{2}w_{j}^{2}\right).$$

Using Bayes's rule, we can define the posterior distribution

(3) $$\begin{array}{c} p\left(w\;|\;t,\;\alpha ,\;\sigma^{2}\right)=\frac{p\left(t\;|\;w,\;\sigma^{2}\right)p\left(w\;|\;\alpha \right)}{p\left(t\;|\;\alpha ,\;\sigma^{2}\right)}, \end{array}$$

which is a Gaussian with mean *μ* = (**F** ^*T*^ **F**+*σ* ^2^ *α* **I**)^−1^ **F** ^*T*^ **t** and covariance matrix Σ = *σ* ^2^(**F** ^*T*^ **F** + *σ* ^2^ *α* **I**)^−1^, where **I** is the identity matrix, **F** a matrix with each row corresponding to a training example in feature space, and **t** a column vector of true labels (classification) for all corresponding training examples. As a result, our hyperplane will have the form *μ* ^*T*^ **f**. This solution is equivalent to a penalized least square estimate *E*(**w**) = (1/2*σ* ^2^)∑_*i*=1_^*N*^(*t*_*i*_ − **w** ^*T*^ **f** ^*i*^)^2^ + (*α*/2)∑_*j*=1_^*n*^ *w*_*j*_^2^ [16]. Regression parameters (*σ* ^2^ and *α*) are tuned with an automatic, iterative procedure [7].

### 3.4. Linear Support Vector Machine

In P300 BCI research, the linear support vector machine (SVM) is regarded as one of the more accurate classifiers [10, 17]. The principal idea of a linear SVM is to find the separating hyperplane, between two classes, so that the distance between the hyperplane and the closest points from both classes is maximal. In other words, we need to maximize the margin between the two classes [18]. Since it is not always the case that the two classes are linearly separable, the linear SVM idea was also generalized to the case where the data points are allowed to fall within the margin (and even are on the wrong side of the decision boundary) by adding a regularization term. For our analysis, we used the method based on linear least squares SVM [19] to solve the minimization problem min _**w**,*b*,**e**_((1/2)**w** ^*T*^ **w**) + *γ*∑_*i*=1_^*N*^ *e*_*i*_^2^ with respect to *y*_*i*_(**w** ^*T*^ **f** ^*i*^ + *b*) = 1 − *e*_*i*_, *i* = 1,…, *n*, where **f** ^*i*^ corresponds to the training points in the feature space, and *y*_*i*_ is the associated output (+1 for the responses to the target stimulus and −1 for the nontarget stimulus). The regularization parameter is estimated through a line search on cross-validation results.

### 3.5. Nonlinear Support Vector Machine

Here, we used a support vector machine with the Gaussian radial-basis function *K*(**f** ^*i*^, **f** ^*j*^) = exp (−*γ*||**f** ^*i*^ − **f** ^*j*^||^2^), *γ* > 0, as a kernel. In our experiment, we opted for the SVMlight package [20]. The SVM's outcome, for a new sample, is a value for *y*(**f**, **w**, *b*) = ∑_*i*=1_^*n*^ *w*_*i*_ *y*_*i*_ *K*(**f**, **f** ^*j*^) + *b*, where **f**_*j*_ are the support vectors chosen from the training set with known class labels *y*_*i*_ ∈ {−1,1}, and where *w*_*i*_ are Lagrange multipliers. The sign of *y*(**f**, **w**, *b*) estimates the class the sample **f** belongs to. For our nSVM classifier, a search through pairs (*C*, *γ*) (where *C* is the regularization parameter and *γ* the kernel parameter) was performed using a 5-fold cross-validation on the grid (*C*, *γ*):[2^−5^, 2^−2^,…, 2^16^]×[2^−15^, 2^−12^,…, 2^6^].

### 3.6. Method Based on Feature Extraction

Another linear classifier used in P300 BCI research [13] relies on the one-dimensional version of the linear *feature extraction* (FE) approach proposed by Leiva-Murillo and Artès-Rodriguez in [21]. The method searches for the “optimal” subspace maximizing (an estimate of) the mutual information between the set of projections *Y* = {**w** ^*T*^ **f** ^*i*^} and the set *T* of corresponding labels *t*_*i*_ = {−1, +1}. According to [21], the mutual information between the set of projections *Y* and the set of corresponding labels *C* can be estimated as *I*(*Y*, *C*) = ∑_*p*=1_^*N*_*t*_^ *p*(*t*_*p*_)(*J*(*Y* | *t*_*p*_) − log *σ*(*Y* | *t*_*p*_)) − *J*(*Y*), with *N*_*t*_ = 2 the number of classes, *Y* | *t*_*p*_ the projection of the *p*th class' data points onto the direction **w**, *σ*(·) the standard deviation, and *J*(·) the negentropy, estimated using Hyvärinen's robust estimator [22].

### 3.7. Artificial Neural Network

For comparison's sake, we also consider a multilayer feed-forward neural network (NN) with a single hidden layer and with sigmoidal activation functions, which is proved to be a universal approximator [23]. Thus, our classifier has the form

(4) $$\begin{array}{c} y\left(f,w,b\right)=\overset{M}{\underset{i=1}{\sum }}w_{i}^{2}F\left(\overset{N}{\underset{j=1}{\sum }}w_{ji}^{1}f_{j}+b_{i}\right)+b, \end{array}$$

where *M* is the number of neurons in the hidden layer, with sigmoidal activation functions *F*(*t*) = 1/(1 + exp (*t*)), *N* the number of observed features, **b** = {*b*_1_,…, *b*_*N*_, *b*} and **w** = {*w*_1_^2^,…, *w*_*M*_^2^, *w*_11_^1^,…, *w*_*NM*_^1^} sets of thresholds and weight coefficients, respectively. The latter were optimized using a training procedure based on the Levenberg-Marquardt back propagation method, where the desired outcome of the neural network was set to +1 or −1 (target or nontarget), depending on the class the individual training example belongs to. Since such a network has *NM* + 2*M* + 1 parameters to be trained, it can easily overfit the training data in the case of a large number of features (*N*), and a large number of hidden layer neurons (*M*). To avoid this, we performed a 5-fold cross-validation with a line search for the number of hidden neurons *M* = 1,…, 20. The network with the best *M* was further retrained on the whole training set.

## 4. Results

 The data was recorded during the * online* typing of words/characters (in copy spell and in free spell mode). In order to assess the classification performance of all classifiers considered, we opted for an *offline* analysis, in which case we also evaluated the performance for a smaller amount of intensification sequences *k*. This became possible since our online spelling was performed with 15 intensifications of each row and column for any character to be typed. This also allowed us to construct a larger amount of test data for *k* < 15. This was done by taking combinations of *k* elements from the available 15 responses for each row and column.

**Figure 3:**
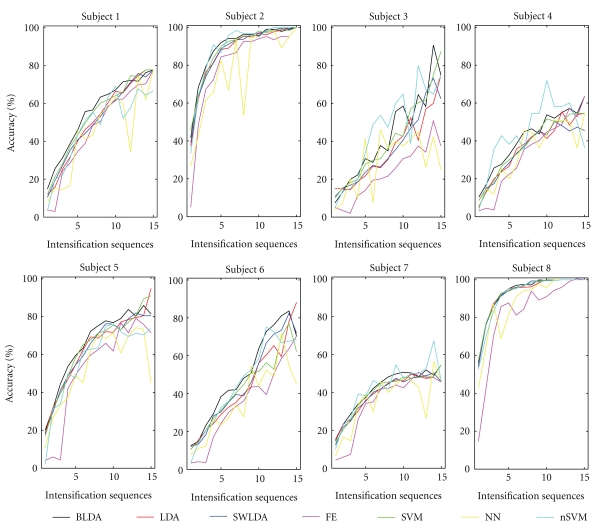
Classification accuracy as a function of the number of intensifications, for every subject, and for all considered classifiers: Bayesian linear discriminant analysis (BLDA), Fisher's linear discriminant analysis (LDA), stepwise linear discriminant analysis (SWLDA), a method based on feature extraction (FE), linear support vector machine (SVM), multilayer perceptron (NN), and Gaussian kernel support vector machine (nSVM).

**Figure 4:**
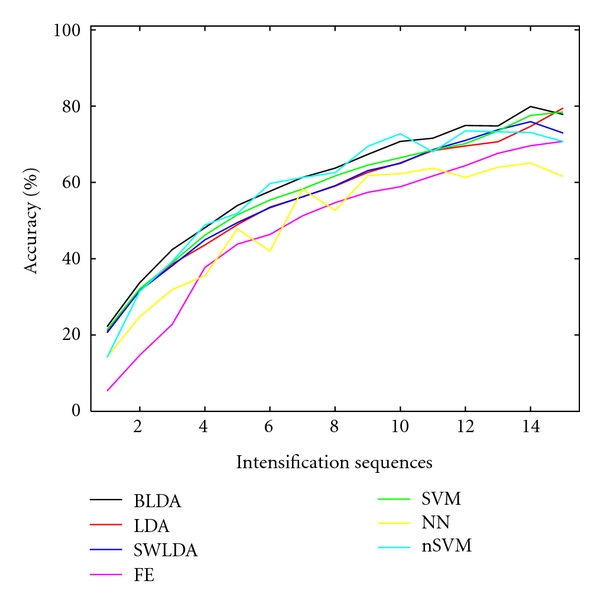
Average classification accuracy as a function of the number of intensifications for all considered classifiers.

The performance results are shown in Figure 3 for each individual patient, and the averaged performance result in Figure 4, averaged over all subjects. In order to verify the statistical significance of the comparison, we used a repeated-measures two-way ANOVA (with “method” and “intensification sequences” as factors) with Greenhouse-Geisser correction (*P* < 0.001 for factor “method”) and with *post hoc* multiple comparison based on Turkey LSD test for pairs of all methods. We found that the accuracy of a BLDA in general is significantly (*P* ≤ 0.02) better than that of any other classifier except the Gaussian kernel SVM (nSVM versus BLDA has *P* = 0.227), since the later, for some subjects, and for some numbers of intensifications *k*, yielded on average better results. Both the linear and nonlinear SVM's (for which the results do not show any significant difference) were second best. As for SWLDA and LDA, which ranked third, SWLDA performs slightly better, but not in a significant way. The worst results are obtained for the feature extraction (FE) method and the multilayer feed-forward neural network (NN).

**Figure 5:**
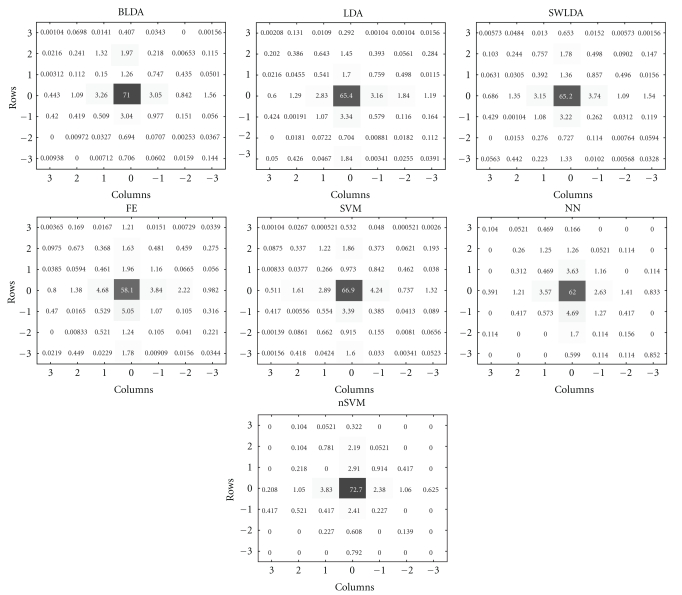
Distribution (percentage with respect to all typed characters) of the P300 speller outputs for *k* = 10 intensifications. Cells with zero coordinates correspond to correctly spelled characters, while other cells show the results of mistyping. The coordinates of those cells indicate the relative positions of the mistyped and intended characters. The presented results are for the Bayesian linear discriminant analysis (BLDA), the Fisher's linear discriminant analysis (LDA), the stepwise linear discriminant analysis (SWLDA), a method based on feature extraction (FE), the linear support vector machine (SVM), the multilayer perceptron (NN), and the Gaussian kernel support vector machine (nSVM).

**Figure 6:**
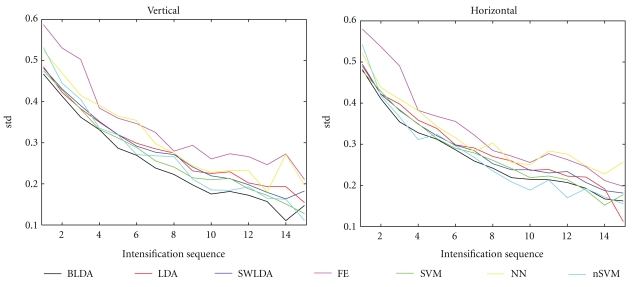
Standard deviations of the vertical distance (left panel) and horizontal distance (right panel) between the typed and desired characters, as a function of the number of intensifications, for each considered classifier: Bayesian linear discriminant analysis (BLDA), Fisher's linear discriminant analysis (LDA), stepwise linear discriminant analysis (SWLDA), a method based on feature extraction (FE), linear support vector machine (SVM), multilayer perceptron (NN), and Gaussian kernel Support Vector Machine (nSVM).

We have also analyzed the distribution of the erroneously typed characters (see Figure 5). We have found that, for all classifiers, the misclassifications mostly occur for either a row or a column in close proximity to the ones of the intended characters (represented at the center of the plot). To investigate any possible differences in the error distributions for each of the considered classifiers, we computed the horizontal (for the columns) and the vertical (for the rows) standard deviations (std) between the typed and the intended characters, and plot this as a function of the number of intensifications (Figure 6). The BLDA classifier for the case of the rows and BLDA together with nSVM for the case of the columns yield, in general, the smallest std, suggesting that those classifiers lead to less wrong answers. In order to verify the statistical significance of the comparison, we used a repeated-measures three-way ANOVA for std using the following factor levels: “method” (with further * post hoc* multiple comparison of all pairwise combinations of classifiers), “direction” (with two levels for this factor, corresponding to rows and columns), and “intensification sequences” (15 levels). We found that the distribution of mistakes around the intended character, based on BLDA, is, in general, significantly (*P* ≤ 0.03 for factor “method”) smaller than for any other classifier, except for nSVM (nSVM versus BLDA has *P* = 0.0829). This suggests that the BLDA, in general, not only yields a better accuracy, but also leads to a smaller divergence in mistakes. We also observe that the vertical standard deviation is in general smaller than the horizontal one (*P* ≤ 0.05 for factor “direction”), particularly for the most accurate classifiers and, especially, after more than 5-6 intensification sequences. For example, for BLDA (fixing this level of factor “method” in previous model), this difference is significant with *P* ≤ 0.02.

## 5. Discussion

Our comparison indicates that, in general, nonlinear classifiers perform worse or equal to linear ones. This is in accordance with other studies [10–12], which were performed on healthy subjects. This could be due to the tendency of nonlinear classifiers to overfit the training data, leading to an inferior generalization performance. It is mostly relevant for the multilayer feed-forward neural network, since the kernel SVM is known to properly deal with high dimensional data and small training sets [18]. In our study, the Gaussian kernel SVM generates a result that is not significantly different from its linear counterpart, but at the expense of an exhaustive grid search. From this, we recommend a linear classifier for a P300 spelling systems for patients, also since, to support its *online* applicability, we have to minimize the classifier's training time.

Among all classifiers the Bayesian linear discriminant analysis (BLDA) yields superior results, with the SVM as the second best, at least for the group of patients considered in our comparison. While a SVM is constructed so as to maximize a margin between the two classes, the BLDA tries to maximize the probability of having training data with the correct class labels. Since both classifiers depend on some regularization parameters, their optimal choice increases the generalization accuracy. This optimization enables us to achieve better results for the P300 speller based on SVM and BLDA. While in SVM, the parameter optimization is done with a search through a discrete set of parameters, in the framework of a cross-validation (thus, depending on the search algorithm, and the resolution of the discretization), BLDA includes a self-adjustment of its parameters via an automatic, iterative procedure. On the other hand, BLDA relies on assumed distributions of the classification errors and of the used parameters.

From the obtained classification results, we observe that different classifiers lead to different accuracies. On the one hand, this shows the necessity to properly choose the classifier for the intended P300 BCI application. But on the other hand, this diversity in results could be turned into a benefit by combining different classifiers in a co-training approach [15], to improve the classification performance.

For the validation of the performance of the classifiers and their comparison, we used as features the amplitudes of the filtered EEG signals from different electrodes. This led to satisfactory results for healthy subjects (see, *e.g.*, [17]). Nevertheless, the accuracy could potentially be improved by adding other features such as time-frequency ones, from a wavelet transform [24], synchrony between EEG channels [25], and the direction and speed of propagating waves [26].

In our experiments, we used electrodes placed at positions Cz, CPz, P1, Pz, P2, PO3, POz, and PO4, which include the parietal ones for which the P300 component is known to be most prominent, but we also added more posterior positions, as suggested in [7, 27–29] where it was shown that the decoding accuracy increases due to the negative-going component, appearing over the posterior areas, prior to the P300 component. To incorporate this additional early information into the decoding process, we used the interval starting 100 ms after stimulus onset. The negative-going component, called N2 in [30], was shown by these authors to be important for the P300 speller, even if the subject only covertly attended the intended target. Thus, for patients, when experiencing problems with eye gazing, the early negative component recorded over the posterior positions seems to be beneficial.

**Figure 7:**
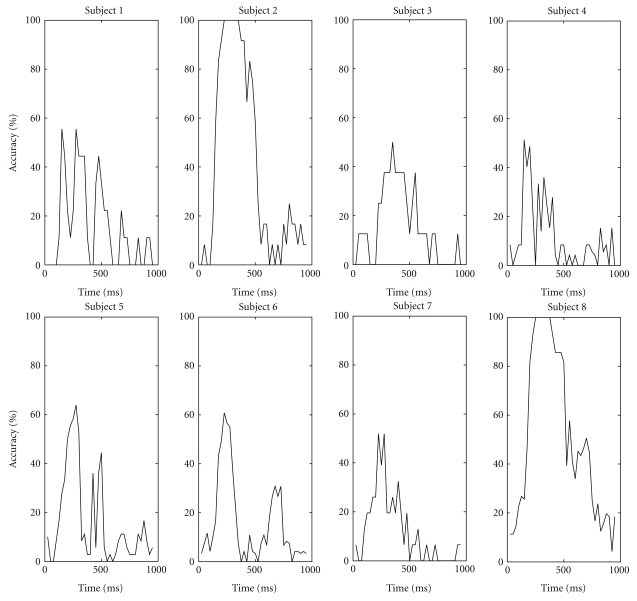
Classification accuracy based on BLDA for every subject as a function of the center of the 50 ms interval from which the features for classification were taken. Consecutive interval centers are spaced by 25 ms.

**Figure 8:**
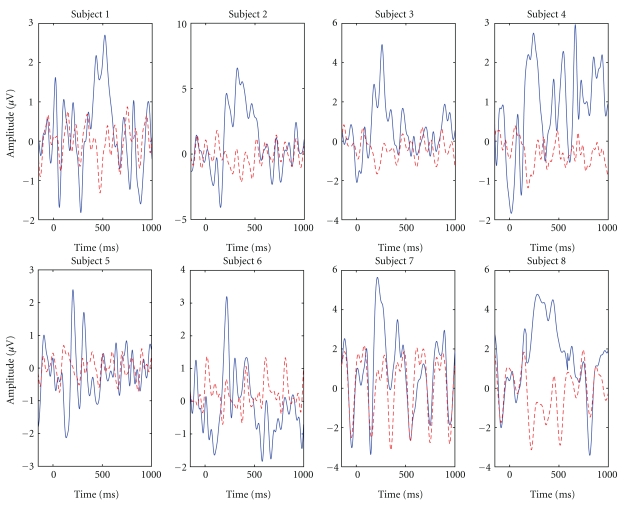
Averaged ERP to target (blue solid line) and nontarget (red dashed line) stimuli for all considered subjects. Baseline correction was performed on the basis of the 150 ms pre-stimulus interval. Zero time corresponds to the stimulus onset. Visible 5 Hz oscillations are due to the stimulation rate.

To validate the added value of the different ERP components into the decoding performance, we estimated the classification accuracy in the P300 speller with 15 intensification sequences and the BLDA classifier, for each patient separately, and for the features taken from 50 ms time intervals (the centers of these intervals were spaced by 25 ms). The classification results are shown in Figure 7, and the averaged ERP waveforms in Figure 8, for electrode POz. The results suggest that the early ERP components should, for some of our patients, also be considered as features for decoding.

The analysis of the distribution of the mistyped characters (Figure 5) suggests that mistakes mostly occur due to a wrongly selected row or column in the typing matrix. Furthermore, we found that the incorrectly typed characters are mostly close to the intended ones. This could, probably, be due to the fact that the subject sometimes gets distracted by the flashing of a column or row adjacent to one containing the intended character. Or, it could be that the intensification of the row/column containing the intended character is immediately preceded or followed by an intensification of an adjacent row/column, leading to a decreased P300 response. As a recommendation, one should try to avoid the consecutive intensifications of adjacent rows/columns. But this is hard to achieve in a row/column paradigm, since in a free spelling mode we do not know *a priori* the character that the subject wants to communicate. Additionally to this, based on the fact that mistakes mostly occur along the row or column containing the desired character, we can try to use some smart scrambling of the intensifications where, instead of a whole row or column, constellations of individual characters, spread over the entire matrix, are intensified. The design of the proper stimulation paradigm as in, for example, [31] is the subject of further research.

Another way to improve the typing performance is by incorporating the detection of the Error Potential (ErrP) [32, 33] into the P300 speller paradigm. The ErrP is evoked when the subject perceives a wrong outcome of the BCI system. When the ErrP is detected, we can take the second most likely character (e.g., the row or the column with the second largest distance to the classification boundary) for correcting the classifier's outcome. Since mistakes are expected to occur in a row or column adjacent to that of the desired character in the matrix (see Figure 5), we can also apply weights to the previous distances (e.g., by inversely relating them to the distance, in the matrix, to the mistyped character).

The typing accuracies achieved by our patients revealed a large variability. While subjects 2 and 8 could achieve an almost perfect typing performance for already *k* = 10 row/column intensifications, subjects 4 and 7 achieved the worst accuracy (around 50% after *k* = 15 intensifications, with a chance level of 100/36 = 2.7%). As can be seen from Table 1, the latter subjects suffered from some form of motor aphasia (as was also the case with three of the four subjects excluded from the classifier comparison study because of bad classification performance (see Section 2.3)). Motor aphasia is known to deteriorate the visual verbal P300 latency more than the visual nonverbal one [34], possibly explaining the inferior performance achieved with these patients. The effect on the P300 speller should be examined further in a study specifically designed for motor aphasia patients.

## 6. Conclusions

We have compared five linear and two nonlinear classifiers in a P300 BCI speller tested on stroke and ALS patients. We have found that the BLDA classifier performs the best, followed by the (non)linear SVM. These results could be helpful to decide what classifier to use for stroke and ALS patients. Finally, we also listed and discussed a number of recommendations for adjusting the P300 speller paradigm to stroke and ALS patients.

## Figures and Tables

**Table 1:** Information about the patients.

Patient ID	Age	Gender	Diagnosis
Subject 1	43	M	Amyotrophic lateral sclerosis. Moderate bulbar palsy. Severe weakness of upper and lower limbs and spasticity in lower limbs.
Subject 2	51	M	Right MCA stroke with hypertension (stage II) and mild left hemiparesis.
Subject 3	58	M	Spontaneous SAH and secondary intracerebral hemorrhage in the right hemisphere with hypertension (stage III) and severe left hemiparesis.
Subject 4	54	F	Left MCA stroke with mild motor aphasia and right hemiparesis.
Subject 5	52	M	Posterior circulation stroke. Right hemiparesis with dysarthria.
Subject 6	54	M	Left MCA stroke with right hemiparesis and motor aphasia.
Subject 7	36	M	Acute left MCA stroke with partial motor aphasia, right hemisensory loss.
Subject 8	65	M	Right MCA stroke with hypertension (stage III) and mild left hemiparesis.
